# Navigating Diabetes in Pregnancy: Critical Approaches to Mitigate Risks and Improve Outcomes for Mother and Child

**DOI:** 10.3390/metabo15030180

**Published:** 2025-03-06

**Authors:** Zoe Paige Garvey, Abhishek Gupta, Nicole Taylor, Mahesh Thirunavukkarasu, Nilanjana Maulik

**Affiliations:** Molecular Cardiology and Angiogenesis Laboratory, Department of Surgery, University of Connecticut School of Medicine, Farmington, CT 06030, USA; zgarvey@uchc.edu (Z.P.G.); abhgupta@uchc.edu (A.G.); nictaylor@uchc.edu (N.T.); mthirunavukkarasu@uchc.edu (M.T.)

**Keywords:** gestational diabetes mellitus, type 1 diabetes, type 2 diabetes, telemedicine, insulin resistance, lifestyle modification, patient education, metformin, glucose monitoring, antihyperglycemic agents

## Abstract

With the increasing prevalence of diabetes and its growing impact on maternal and fetal health, management during pregnancy has become critical. This review describes the pathophysiology of insulin resistance during pregnancy, adverse outcomes correlated with diabetic pregnancies, and current management strategies. We investigate two leading approaches to managing pregnant patients with diabetes—lifestyle intervention and drug treatment. Lifestyle intervention, including dietary counseling, exercise regimens, patient education, and self-administered blood glucose monitoring, has demonstrated promising results in the management and prevention of gestational diabetes mellitus (GDM). Early intervention and treatment of at-risk patients have been critical for positive outcomes. Drug treatment, focusing on the utilization of insulin, insulin analogs, and antihyperglycemic agents has shown efficacy in achieving glycemic control and improving maternal and neonatal outcomes. These findings indicate that a combination of early lifestyle intervention and targeted drug treatment yields the most benefit in managing diabetes in pregnancy. To augment treatment, continuous glucose monitoring and telemedicine have become valuable tools in managing diabetes during pregnancy. Future research should aim to develop more effective antihyperglycemic agents, improve telehealth accessibility, and enhance preconception care for women at risk of developing GDM. By addressing these areas, we can significantly reduce the adverse outcomes associated with diabetes in pregnancy and improve overall maternal and fetal health.

## 1. Introduction

The global incidence of diabetes was estimated to be 828 million of the world’s population as of 2022 [1]. The epidemiological transition of the global population to living longer than ever before while also leading more sedentary lives are seen as some of the major drivers of this increase [2]. This growing global burden of diabetes puts more of the population at risk of the sequelae of diabetes, which include kidney failure, coronary artery disease, and lower limb amputation [3]. Furthermore, it makes pregnancy a high-risk endeavor for a significant portion of the global population. It is well known that insulin resistance and diabetes are risk factors in pregnancy and significantly impact both maternal and fetal outcomes. This includes increased mortality, morbidity, and an enhanced predisposition in both mother and offspring to develop certain lifelong medical conditions. The literature clearly demonstrates that diabetes during pregnancy complicates the outcomes for both mothers and newborns. Offspring face increased risk of developing type 2 diabetes mellitus and in later stages are at risk of developing coronary artery disease [4]. This self-perpetuating situation further contributes to the already growing global diabetic population [1].

Gestational diabetes mellitus (GDM) creates greater health risks for the mother and offspring in the short term due to the various complications that can arise. These complications include macrosomia and shoulder dystocia, which can lead to emergent cesarean delivery and increased maternal morbidity and mortality [5]. Attempts to manage the complications of GDM fall into two camps: lifestyle intervention and medical management. Lifestyle modifications, including diet and exercise with proper education and glucose monitoring, have been championed in some of the literature as effective means of addressing GDM. However, in the context of a growing population with underlying health conditions and factors predisposing to diabetes at baseline, reliance on medical management with insulin and the off-label usage of glucose-lowering agents/disease-modifying therapies, including glyburide and metformin, has become increasingly common to help manage this condition. Despite significant efforts by the medical community and healthcare providers to identify and address diabetes and insulin resistance during pregnancy, the prevalence of GDM has continued to increase by more than 2% between 2016 and 2020 in the United States [6]. It is estimated that in 2013, nearly 16.7% of live births globally had some form of hyperglycemia complicating their pregnancy [7]. This is particularly alarming, as patients who develop GDM have up to a 71% increased risk of developing type 2 diabetes mellitus (T2DM) in the future [8,9,10]. Considering these statistics within the context of the increasing disease burden that diabetes poses for the global population, the need for greater management of diabetes during pregnancy becomes all the more evident.

In this review, we seek to describe the mechanisms underlying GDM to demonstrate the adverse consequences that such a condition poses to all pregnancies and outline the most effective means of preventing and managing insulin resistance in pregnancy to improve maternal and fetal outcomes. Despite pregestational and gestational diabetes differing in pathophysiology, we use an integrated approach in discussing these conditions due to their overlapping treatment recommendations. Section I outlines the pathophysiology leading to increased insulin resistance, how a greater risk of diabetes among the general population at baseline further potentiates these risks, and the adverse effects that can occur to mothers and children, as well as the current prevalence of such events. Section II presents the approaches to lifestyle intervention and medical management of diabetes in pregnancy. Section III proposes a generalized, combined therapeutic approach from the different management styles presented in Section II while also highlighting the importance of patient engagement and proposing the utilization of modalities such as telehealth to help accomplish this. Finally, Section IV details areas of further inquiry and research needed to better address this problem, including increased accessibility for patients regarding screening and education, as well as developing more effective glucose-lowering/disease-modifying therapies approved for use in pregnancy.

The methodology for collecting literary sources utilized searching the PubMed database with the following keywords: gestational diabetes mellitus; type 1 diabetes; type 2 diabetes; telemedicine; insulin resistance; lifestyle modification; patient education; metformin; glucose monitoring; antihyperglycemic agents. Search results were evaluated based on their clinical relation and application to the incidence, pathophysiology, management, and outcomes of metabolic syndrome in pregnancy for inclusion in this article. The literature that extended beyond the scope of this article including the management of diabetes outside of pregnancy was not included.

## 2. Section I: Diabetes and Pregnancy

Pregnancy induces a wide variety of physiological changes, the vast majority of which are meant to support fetal development and are reversible. Insulin resistance during the second and third trimesters of gestation is one of these expected changes and serves an important purpose in successful fetal growth. This section will define the physiological effects of insulin resistance in both uncomplicated pregnancies and those complicated by diabetes, as well as delineate the adverse outcomes of diabetic pregnancies for both mother and child.

**Figure 1:**
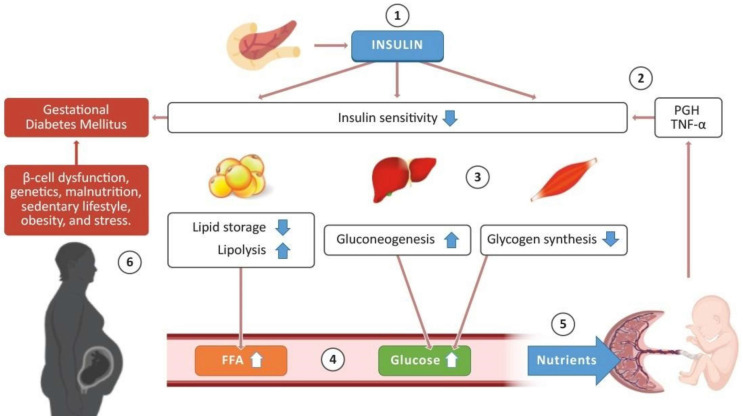
The flow diagram represents the metabolism of glucose–insulin during pregnancy. (1) Insulin is released from pancreas and regulates glucose level in the blood stream. (2) In a normal pregnancy, the placenta generally produces placental growth hormone (PGH) and releases inflammatory cytokines; (3) consequently, adipose tissue activates lipolysis. (4) This increases fatty acids (FFAs) and glucose in the blood stream, and (5) these are required for the development of the placenta and for the fetus to grow healthy, (6) which leads to the development of gestational diabetes mellitus in some pregnant women. Blue down arrow represents downregulation and white arrow represents upregulation. Adapted from Dennise Lizárraga et al. and reproduced with permission [11].

**Figure 2:**
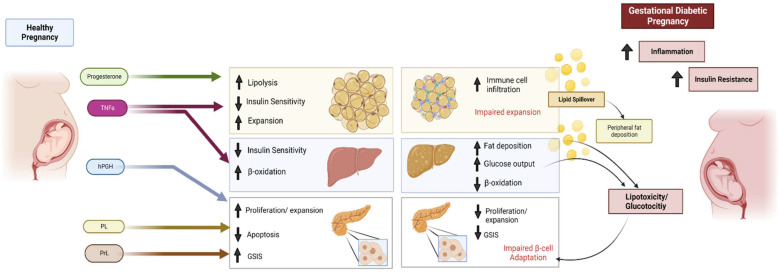
Metabolic adaptations of late gestation and the impact of gestational diabetes. ↑ increased; ↓ decreased; TNFa: tumor necrosis factor-alpha; hPGH: human placental growth factor; PL: placental lactogen; PrL: prolactin; GSIS: glucose-stimulated insulin secretion. Adapted from Brittany L. Moyce Gruber et al. and reproduced with permission [14].

Insulin is a necessary hormone for glucose uptake within the body, specifically within the insulin-sensitive cells of the liver, muscle, and adipose tissue [11]. It is a primary regulator of the body’s energy supply. Insulin binding to insulin-dependent glucose transporter-4 (GLUT4) receptors in white adipose tissue and striated muscle stimulates further GLUT4 expression on cell membranes, glucose oxidation, adipose lipoprotein lipase activity, and synthesis of free fatty acids and triacylglycerols. Overall, it facilitates carbohydrate, lipid, and protein metabolism [12]. Insulin resistance is characterized by a decreased responsiveness of organs and cells within the body to glucose [13]. Consequently, less glucose is taken up into cells. This change is favorable for the fetus during the second trimester, as it increases its glucose demand for growth [11]. Glucose can cross the placenta, but insulin cannot; thus, the increasing insulin resistance of the mother physiologically benefits the fetus by increasing the amount of glucose available to it [13]. To understand the basis for insulin resistance in pregnancy, it is important to contextualize it as a result of greater hormonal changes occurring within the mother due to pregnancy. The placenta plays a unique role during pregnancy. Figure 1 summarizes insulin metabolism in diabetic pregnancy [11]. Figure 2 contrasts states of insulin resistance in healthy and diabetic pregnancies [14].

**Figure 3:**
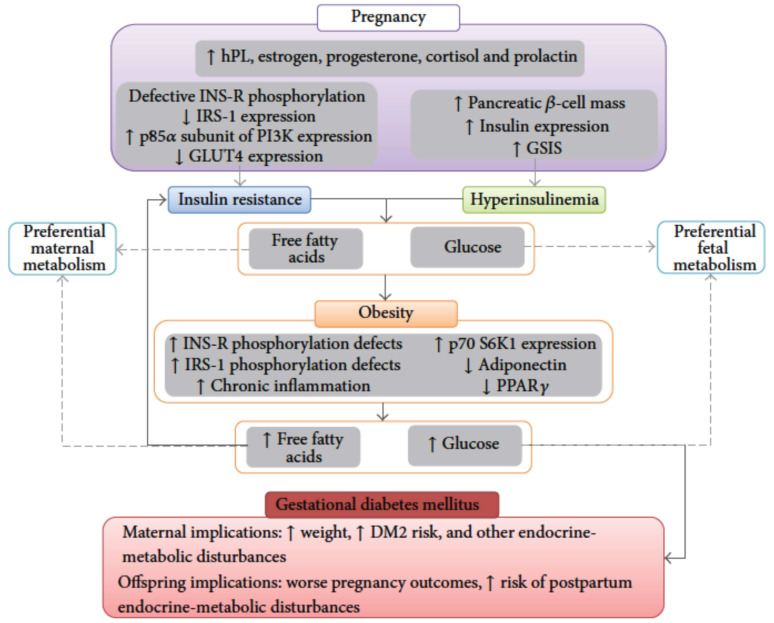
The flow diagram shows the molecular mechanisms underlying insulin resistance in normal pregnancy physiology and gestational diabetes mellitus. During states of insulin resistance, there is increased secretion of human placental lactogen (hPL), estrogen, progesterone, cortisol, and prolactin. They generally favor the increased release of free fatty acids, which are predominantly metabolized by mothers, allowing for the shunting of glucose towards fetal metabolism. DM2: type 2 diabetes mellitus; GSIS: glucose-stimulated insulin secretion; hPL: human placental lactogen; INS-R: insulin receptor; IRS-1: insulin receptor substrate-I; PI3K: Phosphoinositide 3-Kinase; GLUT4: Glucose transporter 4; p70 S6K1: P70-S6 Kinase 1; PPARy: peroxisome proliferator-activated receptor; ↑ represents increased; ↓ represents decreased. Adapted from Joselyn Rojas et al. and reproduced with permission [13].

Maternal metabolism exhibits increased sensitivity to insulin during pregnancy due to increased glucose demands and hyperphagia, which are necessary for fetal and placental development. These changes lead to hypertrophy and hyperplasia of the pancreatic β-cells, which increases insulin secretion at “physiological concentrations of glucose” [15]. During the second trimester, human placental lactogen (hPL) secretion increases from the placenta, and its antagonistic effects on insulin and its lipolytic effect are the major catalysts for the insulin-resistant state in maternal metabolism, which peaks in the last half of pregnancy [11]. The increase in hPL levels, combined with elevated levels of estrogen, progesterone, cortisol, and prolactin during pregnancy (as seen in Figure 3), decreases insulin sensitivity and increases gluconeogenesis from the liver, lipolysis from adipose tissue, and proteolysis from muscle tissue due to a decreased insulin response [13,14]. This results in a shift in the maternal metabolism to a catabolic state that is glucose-sparing, effectively limiting maternal glucose uptake and enhancing the nutrient transfer of glucose to the developing fetus [16]. Such a state can induce abnormal gestational insulin resistance, including gestational diabetes, the onset of diabetes mellitus, or further exacerbation of pre-existing diabetes mellitus, especially in patients who are overweight at baseline [16].

Factors contributing to the hyperglycemic state in certain patients include genetics, metabolic conditions, and inflammatory states as demonstrated in Table 1 [11,17]. Additional factors, specifically regarding genetic predisposition, are currently the subject of further research [17]. The impact of such conditions is harmful not only to the mother but also to the fetus.

Regarding maternal health, pregnant mothers who develop GDM are much more likely to progress to T2DM after pregnancy. Both Aroda et al. and Ratner et al. found that women who experience GDM are 71% more likely to develop T2DM in the future, with the latter study maintaining that this heightened risk persists long after the completion of pregnancy [8,9,10]. Additionally, pregnancies complicated by diabetes are more likely to require cesarean delivery [18,19]. Mortality rates have also been found to be higher for pregnant mothers with pregestational or pre-existing diabetes. Tavera et al. found that compared to women without pregestational diabetes, women with pregestational (type 1 or type 2) diabetes were three times more likely to experience cardiac arrest or “in-hospital death”, and women with GDM had no heightened risk for cardiac arrest and were actually 38% less likely to experience “in-hospital death” compared to the control population [20]. Furthermore, according to Negrato et al., pregnant women with type 1 diabetes (T1DM) have a death rate significantly higher than that of the “general population” [18]. Finally, women with diabetes or GDM are at a higher risk of developing pre-eclampsia and eclampsia, as well as Hemolysis, Elevated Liver enzymes, and Low Platelets (HELLP) syndrome [21,22]. All these conditions have been associated with significant maternal and fetal morbidity and mortality [18]. Just as the mother is negatively affected by the sequelae of diabetes/GDM, so is the fetus.

**Figure 4:**
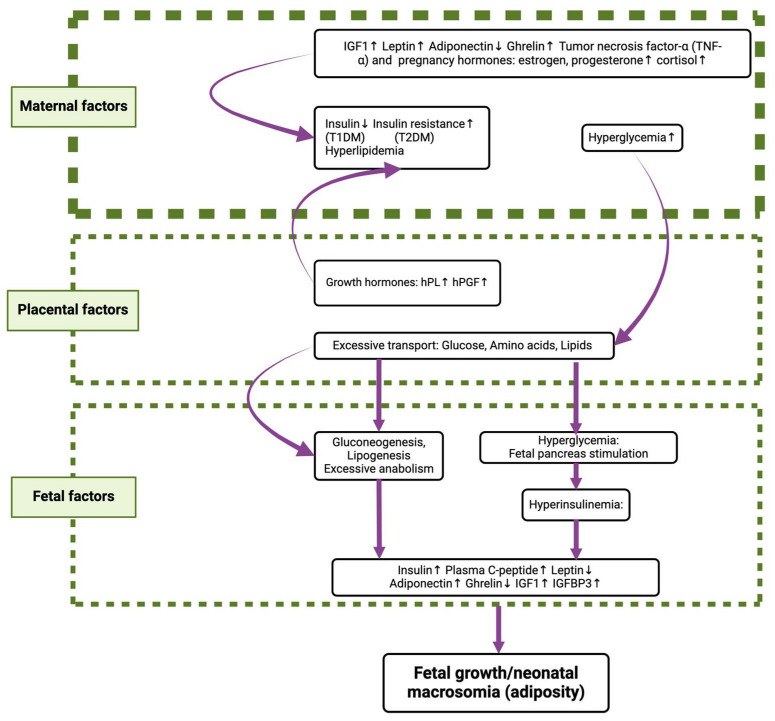
This figure denotes factors affecting fetal growth in diabetic pregnancies. *Insulin resistance* induces glucose intolerance, resulting in hyperglycemia. This, in turn, causes maternal hyperglycemia by hyperinsulinemia, which in turn reduces fetal blood glucose levels but also increases fetal adipose tissue and enhances growth. In addition, placental growth hormones facilitate fetal gluconeogenesis and lipogenesis, further contributing to enhanced fetal growth. IGF1: insulin-like growth factor-1; T1DM: type 1 diabetes mellitus; T2DM: type 2 diabetes mellitus; hPL: human placental lactogen; hpGF: human placental growth factor; IGFBP-3: insulin-like growth factor-binding protein 3. ↑ represents increased; ↓ represents decreased Adapted from Asher Ornoy et al. and reproduced with permission [16].

Babies born to mothers with diabetes mellitus or GDM are at an increased risk of having macrosomia and generally higher birth weight [23,24], as summarized further in Figure 4 [16]. Adane et al. noted that the likelihood of macrosomia is even higher when mothers have GDM compared to when they have pregestational diabetes. Neonates born to mothers with GDM are at an increased risk of having malformations of the cardiac and/or central nervous systems, including “transposition of the great arteries, septal defects, neural tube defects, and caudal regression syndrome” [25,26] as well as circulatory issues, including “rheumatic fever, hypertensive disease, ischemic heart diseases, disease of pulmonary circulation, and other forms of heart disease, cerebrovascular disease and diseases of arteries, veins, and other diseases of the circulatory system” [27]. Many studies and reviews have proposed a potential increased risk of cognitive impairments in such babies, though the links have yet to be proven [25,26,28,29]. Finally, there is an increased rate of hyperinsulinemic hypoglycemia in newborns, neonatal respiratory distress syndrome, stillbirth, and perinatal mortality [23,30,31].

**Figure 5:**
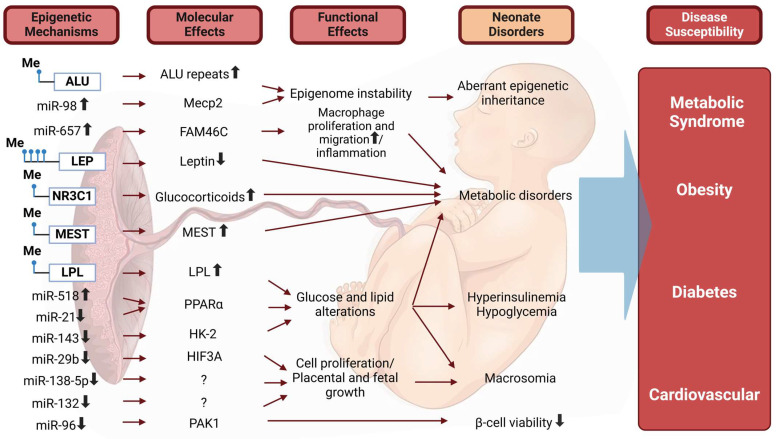
Alterations in DNA methylation and miRNA expression in the placenta from women with GDM will alter the expression and function of genes involved in metabolic and cellular pathways. As a result of these changes, the neonate is susceptible to hypoglycemia, hyperinsulinemia, macrosomia, metabolic disorders, and reduced variability of pancreatic β-cells. This susceptibility may later lead to the development of metabolic syndrome, obesity, diabetes, and cardiovascular complications. ↑ represents increased; ↓ represents decreased Adapted from Dennise Lizárraga et al. and reproduced with permission [11]. Created with BioRender (https://BioRender.com).

Exposure to the abnormal insulin-resistance states detailed above during gestation has also been shown to impact the long-term health of the child. This includes a predisposition toward childhood obesity and an increased risk of early-onset cardiovascular disease. These offspring are also more likely to develop T1DM or T2DM later in life, with offspring born to mothers with GDM more likely to develop impaired glucose tolerance between the ages of 10 and 14 [32]. Limited evidence suggests the potential for long-term respiratory distress. As Azad et al. noted, “delayed lung maturation and increased risk of respiratory distress syndrome (RDS) have been consistently observed among infants born to mothers with diabetes, and these findings are recapitulated in rodent models of diabetes in pregnancy” [33]. Figure 5 highlights the short- and long-term health consequences of diabetic pregnancies on the fetus due to alterations in DNA methylation and miRNA expression [11].

To date, the scientific literature outlines a clear understanding of not only the pathogenesis of diabetes in pregnancy but also the unfavorable outcomes. As further detailed in Section II, the literature has also identified ways to manage such conditions through lifestyle changes and pharmacological therapy. However, despite efforts to manage these conditions, abnormal insulin resistance in pregnancy continues to be a prevalent problem. The diagnosis of GDM increased by over 2% between 2016 and 2020 in the United States [6], and estimates indicate that nearly 16.7% of live births globally in 2013 had some form of hyperglycemia complicating their pregnancy [7], suggesting a greater need for discussion and research on the topic.

## 3. Section II Treating Diabetes During Pregnancy—The Two Perspectives

There are two primary approaches to managing pregnancies complicated by diabetes: drug treatment and lifestyle intervention. Drug treatment involves the implementation of insulin therapy, utilizing either human insulin, insulin analogs, or an expanding array of antihyperglycemic agents to achieve glycemic control, thus minimizing the effects of recurrent hypoglycemia and hyperglycemia. Lifestyle intervention is defined as a collection of dietary modifications, exercise plans, education, and blood-glucose self-monitoring undertaken in order to address the underlying health conditions that lead to diabetes and gestational diabetes [34].

### 3.1. Section II A: Lifestyle Intervention

Proper lifestyle intervention alone can dramatically decrease the risk of developing GDM and mitigate adverse outcomes associated with pregnancies complicated by diabetes. Studies have determined that 70–90% of women diagnosed with GDM can control their condition through this method alone, given the proper selection of criteria. Lifestyle intervention is multifaceted with differing approaches to each aspect, yet the most effective of these approaches account for four key factors [35]. As defined by Guo et al., these factors include identifying those most at risk of developing GDM, initiating intervention early (prior to 20 weeks gestation), enforcing a minimum exercise standard (50–60 min of moderate intensity activity twice weekly), and controlling weight gain during gestation [36]. While weight gain is expected in pregnancy, and guidelines have been created in the past, the current literature suggests that reducing excessive gestational weight gain lies in personalizing the weight gain goals to the patient to better target the prevention of negative maternal and fetal outcomes. This is further evidenced by previous attempts to outline gestational weight gain goals of obese patients that have been found to be suboptimal in regard to improved outcomes [37]. Numerous works have confirmed that early risk identification and intervention with education, dietary advice, instructions to check capillary blood glucose, and pharmacotherapy with insulin or metformin if indicated improve maternal and neonatal outcomes in a diabetic context [38]. Studies also suggest that engagement in lifestyle modification of diet and exercise pre-pregnancy is the best way to prevent GDM and manage diabetes once gestation begins. Specifically, these studies utilized individualized calorie-restricted diets based on body weight and exercise regimens ranging from a minimum of 150 min per week up to 60 min per day [37,39,40]. While the evidence is promising, variability in the compliance and feasibility of these interventions in the clinical setting pose a barrier to applying such a method universally. Natural barriers to lifestyle intervention are prone to manifest during pregnancy. For example, physical activity can become limited due to concerns about harming the fetus. Biological and physiological changes leading to nausea and cravings can undermine adherence to exercise and diet programs. Additionally, social determinants of health including financial resources to pursue diet and exercise programs, healthcare access, transportation, and the safety of the living environment can all act as barriers and alter outcomes of such interventions amongst GDM patients. Furthermore, it can be difficult for patients to initiate new lifestyle changes to diet and exercise on top of navigating a pregnancy. Finally, there is a relatively short window after which GDM has been identified for interventions to be implemented and positive changes to be realized [39].

Notably, a minority of the scientific literature claims that lifestyle intervention does not have a significant effect on a range of maternal and offspring outcomes [41,42]. An Australian trial of women with a body mass index (BMI) of 25 kg/m^2^ at 10–20 weeks of gestation determined that lifestyle intervention with dietary and weight management counseling did not significantly impact gestational weight gain. Counseling in this study was administered over three in-person sessions and three telephone calls with either a dietician or research assistant [43]. Another study showing non-significant results from lifestyle interventions with dietary and exercise counseling, personalized meal plans, and recommendation for 30 min of low-impact aerobic activity three times weekly conceded that the exercise component should have been more rigorous, and their subjects had a lower BMI than those chosen in other studies [44]. Brown et al. stated that there is “no clear difference” in the risk of developing hypertension during pregnancy, needing cesarean delivery, perinatal death, or developing T2DM 4.5–10 years after pregnancy in their study comparing the outcomes of pregnancies complicated by GDM to a control group [34]. Despite this, they conceded that specific offspring outcomes improved where maternal outcomes did not. Furthermore, no study has been able to demonstrate a negative association between lifestyle intervention and outcomes of pregnancies complicated by diabetes. With the majority of the scientific literature demonstrating improved outcomes through lifestyle intervention, examining and refining specific practices in this field is worthwhile to yield the best results.

Dietary modification has been shown to benefit maternal and offspring outcomes in pregnancies complicated by diabetes [45,46]. One trial illustrated the impact of dietary advice administered by a licensed dietician through two in-person and two telephone counseling sessions, demonstrating that a lifestyle intervention plan consisting of dietary recommendations based on the American Diabetes Association and exercise, consisting of 150 min per week of moderate intensity exercise or a step goal of ten thousand per day, managed to significantly reduce the incidence of GDM in at-risk women despite only a 30% adherence rate to the physical activity plan [42]. Louie et al. designed a trial in which one group of pregnant women followed a low glycemic index (LGI) diet and another a conventional high-fiber diet, ultimately finding both groups to have a lower glycemic index, with the LGI group having a lower birth weight and lower birth centile [47]. Sweeting et al. found that in pregnancies complicated by GDM, a targeted dietary intervention focused on enhancing nutritional quality, irrespective of approach, generally improved “maternal fasting and postprandial glycemia and reduced pharmacotherapy requirements, birth weight, and macrosomia” [48]. There has been an effort in the medical community to establish a diet plan specifically targeted at reducing the risk of developing GDM and preventing the worst outcomes in pregnancies complicated by T1DM and T2DM. One such plan designed by Lende and Rijhsinghani closely follows the dietary guidelines set by the American Diabetes Association (ADA) and has shown positive results [49]. However, another study in which the plan was centered around the Dietary Approaches to Stop Hypertension (DASH) diet (a diet initially designed to combat hypertension but which was found to improve glucose control) noted an effective reduction in the need for insulin and cesarean delivery among women with GDM [46]. Egan and Dunne highlighted the lack of a universally accepted dietary plan aimed at managing diabetes during pregnancy, arguing that this has led to varied results globally [50]. They contended that the most basic plan is outlined in the Dietary Reference Intakes (DRIs), which recommend “a minimum of 175 g of carbohydrate, 71 g of protein, and 28 g of fiber for all pregnant women” [50].

Given that such a wide array of dietary plans could have positively influenced the outcomes of these trials, it would be beneficial to establish the most basic plan (that still clearly yields improved results) as a baseline for healthcare professionals to employ. This would allow for a simple guideline for patients to follow and for medical professionals to establish more targeted plans based on individual needs. Still, when dietary advice is paired with other aspects of lifestyle intervention, the best outcomes are achieved. It has been shown that in women with a history of GDM and/or a pre-pregnancy BMI >/= 30 kg/m^2^, a combination of physical activity and dietary modification can lead to as much as a 39% reduction in GDM incidence among this at-risk population over the course of their pregnancies [51]. In this study, lifestyle interventions consisted of reaching a minimum of 150 min of moderate intensity physical activity per week (defined as activity that makes the participant “at least slightly out of breath and sweaty”) and monitored dietary guidance with trained nutritionists and nurses which avoided weight gain in the first two trimesters and emphasized the consumption of fiber-rich whole grains, fish and low-fat meat products, vegetables, fruits and berries, and unsaturated fatty acids [51]. A study by Mohsenzadeh-Ledari et al. additionally found that pregnant women with metabolic syndrome had lower two-hour postprandial glucose levels when provided with a comprehensive lifestyle intervention program consisting of motivational interviewing, dietary modification of nutrition with meals made up of 20% protein/30% fat/50% carbohydrates, and exercise including bodybuilding, walking, and stretching [51,52]. Studies have shown that exercise alone can also improve outcomes for pregnant women with diabetes or those at risk of developing GDM [37,40].

Physical activity has also been proven to positively impact pregnancies complicated by diabetes and has a particularly significant effect on the prevention of GDM among at-risk women. In their analysis of over 30,000 pregnancies, Juan and Yang found that women who reported any level of physical activity during the pre-pregnancy or early pregnancy period were 30% and 21% less likely to develop GDM, respectively [40]. Guo et al. cited a study that put this number at 55% and 24%, respectively [37]. Furthermore, women who consistently engaged in over 90 min of “leisure-time physical activity” before pregnancy experienced a 46% decrease in their risk for GDM [40]. These results include experimental groups with varying BMIs, but it is worth noting that physical activity is most effective at preventing GDM among women with a BMI of 33 kg/m^2^ or higher [53]. Women who already have GDM can also benefit from physical activity, with one systematic review identifying that studies with a variety of aerobic and resistance exercise programs were all found to improve fasting, postprandial, and HbA1c glucose control [54]. The positive effects of physical activity on the outcomes of pregnancies complicated by diabetes are not limited to preventing and managing GDM. One study focusing on an experimental exercise group composed of pregnant women with T1DM found this group to have fewer instances of cesarean delivery, and their offspring exhibited reduced prevalence of hypoglycemia, hypocalcemia, hyperbilirubinemia, and macrosomia [55]. Furthermore, a review article evaluating studies with varying modalities of exercise, termed leisure-time physical activity, identified a protective effect of physical activity on the development of pre-eclampsia [56]. Notably, women with higher levels of education are more compliant with exercise programs, and education itself can be an effective means of improving outcomes in pregnancies complicated by diabetes [57].

Patient education remains difficult to quantify, yet it is an essential component of treating diabetes during pregnancy. Azzam and El Sharkawy found, after a period of 37 weeks, that women with GDM who attended an instructional targeted health education module on gestational diabetes which included education on the disease, management with blood sugar measurement and insulin administration, and the importance of a high-fiber, low-sugar diet, and daily walking of 30 min had decreased blood glucose levels compared to women with GDM who did not [58]. They also determined that the experimental group experienced lower incidence rates of “polyhydramnios, preterm labor, pregnancy-induced hypertension, premature rupture of membranes, and vaginal infection”, which could be attributed to the education module [58]. A feeling of empowerment through education has also increased satisfaction levels in multiple trials, with increased access to information being highlighted as a positive aspect of treatment among patients [59,60]. The growing telemedicine field is becoming an essential source of information, allowing for increased access to medical professionals and educational materials, which can be particularly helpful amongst rural populations and those who cannot easily access healthcare institutions [61]. Still, with limited trials quantifying the effect of education alone, it remains difficult to disentangle it and examine it outside of other aspects of lifestyle intervention, such as the self-monitoring of blood glucose levels.

The self-monitoring of blood glucose levels is essential in minimizing diabetic complications related to pregnancy. Sweeting et al. found that a combination of dietary advice, self-monitoring of blood glucose levels, and insulin therapy (if required) reduced the incidence rate of macrosomia, decreased the likelihood of a composite of perinatal severe complications (“a composite of death, shoulder dystocia, nerve palsy, and fracture”), and improved maternal health [48]. Innovations in the field of continuous glucose monitoring are heralded by many as essential in improving the outcomes in pregnancies complicated by diabetes. Ringholm et al. contended that the Flash glucose monitoring system is associated with increased patient satisfaction, a reduction in hypoglycemia, and improved HbA1c levels compared to the finger-stick method [62]. They suggested that these results would likely be mirrored by patients with GDM and that trials should be conducted to determine whether this system can improve GDM management. Indeed, a study by Schaefer-Graf et al. demonstrated that the use of a continuous glucose monitor (CGM) system, as opposed to capillary glucose monitoring, produced “an approximately 50% reduction in LGA, neonatal intensive care admissions >24 h, and neonatal hypoglycemia” [29]. Another trial by Murphy et al. also resulted in lower birth weights and decreased risk of macrosomia among the offspring of mothers with pregestational diabetes who used CGM [63]. Increased integration of telehealth and CGM systems has also yielded positive results, as the ability and motivation to upload glucose levels to telehealth apps has led to higher levels of compliance with blood glucose monitoring [60].

Notably, the strongest proponents of lifestyle intervention recommend implementing this method of diabetes management in conjunction with drug treatment [64]. The importance that many place on implementing lifestyle interventions during the earliest stages of pregnancy or even pre-pregnancy underscores the significance of timing for this method of diabetes management. In cases where this window has passed, or to more effectively minimize complications for pregnant women who are most at risk of developing GDM or have pregestational diabetes, drug treatment can help patients achieve better outcomes.

### 3.2. Section II B: Drug Treatment

Insulin therapy is the standard treatment for diabetes, with insulin analogs and antihyperglycemic agents becoming increasingly prevalent in treatment compared to human insulin. Insulin was introduced as a treatment for diabetes mellitus in the 1920s, marking a revolutionary breakthrough in the medical field. Despite this, it quickly became clear that native human insulin injection therapy was a suboptimal method for achieving glycemic control. The significant delay period from normal insulin injection to peak insulin absorption and concentration results in postprandial hyperglycemia. Because insulin levels decrease slowly after this peak, there is a greater chance of late hypoglycemia [65]. Insulin analogs began to be developed in the 1980s, drawing upon research on the chemical synthesis of insulin dating back to the 1960s and the technological advances of protein engineering and biosynthesis [66]. Insulin lispro and Insulin aspart are the two major rapid-acting insulin analogs that emerged in the late 80s and 90s, each demonstrating an ability to reduce glycosylated hemoglobin levels and the frequency of hypoglycemia in patients [67]. In the early 2000s, exploratory research into oral and inhaled insulin analogs and antihyperglycemic agents began, and many in the medical community were optimistic that they could replace prandial insulin in the future [67]. Two such antihyperglycemic agents among contemporary studies have emerged as promising treatments for pregnant mothers: glyburide and metformin.

Glyburide is an antihyperglycemic agent that was first available in European markets in 1969, though its use in the United States was delayed until 1984 [68]. One of the members (Glyburide) of the sulfonylurea class stimulates insulin secretion from the beta cells of the pancreas to lower blood glucose levels [68]. One of the earliest studies to evaluate the effectiveness of orally administered glyburide was published in 2000, finding no significant differences in maternal and perinatal outcomes in patients with GDM receiving glyburide treatment compared to those receiving insulin treatment [69]. In 2004, a clinical study conducted by Kremer and Duff determined that glyburide treatment achieved satisfactory glycemic control in 80% of women unable to do so with dietary intervention alone following the American Diabetes Association diet (30 kcal/kg of ideal body weight) [70]. Despite the positive results of early studies, more recent works have demonstrated some negative associations between oral glyburide treatment and maternal and offspring outcomes. It has been noted that glyburide increases the risk for macrosomia by 2-fold compared to insulin therapy [41] and raised concerns that transplacental glyburide and fetal exposure were possible mechanisms underlying this outcome. Castillo et al. demonstrated a wider array of risks associated with glyburide treatment in pregnant mothers. In addition to an increased risk of macrosomia, they found significant but less frequent occurrences of cesarean delivery, preterm delivery, obstetric trauma, and jaundice [71]. Many recent studies have found that the maternal and offspring outcomes of diabetic mothers treated with metformin compare favorably to those of mothers treated with glyburide [72,73]. Feghali et al. summarized the results of several studies comparing glyburide to insulin and/or metformin treatment [74].

**Figure 6:**
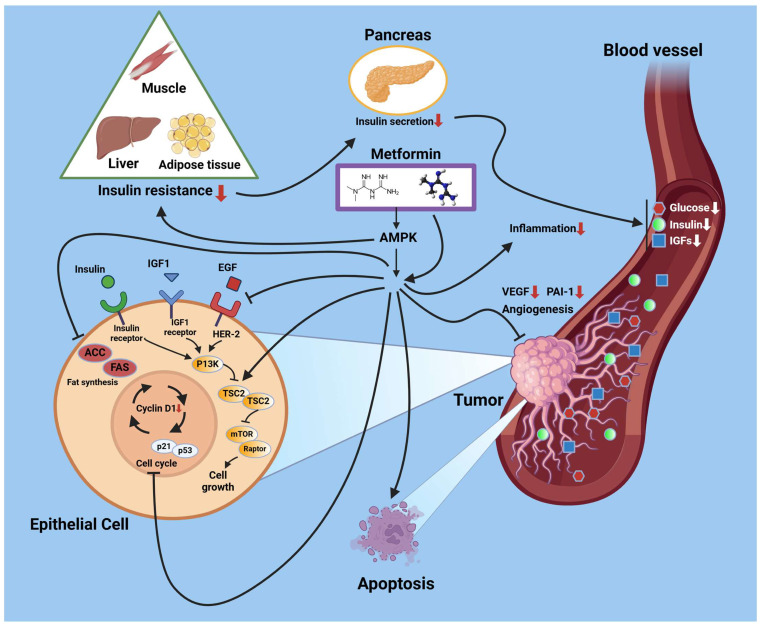
Unique molecular mechanism of metformin in various pathways. Metformin reduces insulin resistance, insulin secretion, glucose blood levels, inflammation, and angiogenesis as well as resulting in a reduction in cell growth and metabolism that mediates its anti-tumor activity. These effects are regulated by both AMPK-dependent or -independent mechanisms that lead to the inhibition of mTOR signaling. (Abbreviations: ACC, acetyl-CoA carboxylase; AMPK, 5′ adenosine monophosphate-activated protein kinase; IGF, insulin-like growth factor; EGF, epidermal growth factor; FAS, fatty acid synthase; PAI-1, plasminogen-activator inhibitor-1; PI3K, Phosphatidylinositol-4,5-bisphosphate 3-kinase; TSC2, tuberous sclerosis 2; mTOR, mechanistic target of rapamycin; VEGF, vascular endothelial growth factor). Adapted from Roberto Romero et al. and reproduced with permission [75]. Created with BioRender (https://BioRender.com).

Metformin (belongs to the biguanide class of antidiabetic drugs) was approved for clinical use in treating diabetes in Europe since the 1970s but was only approved for use in the United States in 1995 [75,76,77]. Metformin, a member of the biguanide class, produces an antihyperglycemic effect through multiple mechanisms including decreasing hepatic glucose production, intestinal absorption of glucose, and increasing insulin sensitivity. It was previously thought that an advantage of metformin treatment is that although it can pass through the placenta, the fetus absorbed a minimal amount, and, therefore, there was no evidence of a negative impact on the fetus [75]. However, meta-analysis has shown that exposure to metformin during pregnancy could lead to smaller neonates that demonstrated increased rates of growth postnatally and consequently higher BMI in childhood [78]. A follow-up study demonstrated an association between metformin use in pregnancy and an increased childhood weight-to-height ratio and waist circumference. As with lifestyle intervention, early treatment appears to be key in achieving the best results through metformin treatment. It also allows for the safest treatment path, as a meta-analysis of the literature on the subject found no increase in fetal malformation risk if metformin treatment begins in the first trimester [76]. Metformin is broadly associated with a reduction in instances of macrosomia in offspring across numerous studies [48,77,78,79]. Other effects on neonatal outcomes observed in metformin trials include reduced incidence rates of low-for-gestational-age babies [79] and lower instances of hypoglycemia [80,81,82]. It is noteworthy that multiple trials have determined that metformin therapy can increase preterm birth rates and lower gestational age at delivery [81,82]. However, other concerns raised regarding the potential impact of metformin exposure on the cognitive development of offspring appear unfounded, as clear evidence of this has yet to emerge [83]. A comprehensive range of improvements in maternal outcomes have been associated with metformin treatment. It has been shown to yield improved glycemic control over a placebo [84], regular insulin [80], and glyburide [73]. Women receiving metformin treatment during pregnancy are less likely to require supplementary insulin [78,79]. Metformin can reduce the likelihood of women with a prior history of GDM developing T2DM [8,9]. Nachum et al. found that women with polycystic ovarian syndrome treated with metformin during pregnancy were significantly less likely to develop GDM than those who were not (31% vs. 3%) [73]. Maternal weight gain is reduced with metformin rather than with insulin treatment [72,79,81]. Metformin combined with regular insulin therapy reduces the need for a cesarean delivery by 30% compared to metformin or insulin treatment alone [84,85]. Supplementing insulin treatment with metformin rather than increasing insulin dosage in mothers with GDM also lowered hospitalization rates and care costs [80]. Finally, metformin treatment reduced the incidence of pregnancy-induced hypertension and pre-eclampsia [86]. Figure 6 outlines the effects of metformin and its signaling pathways [75]. These outcomes, paired with its low cost and high availability, have made metformin a widely used medication in GDM, though social determinants of health may pose barriers to ensuring equal outcomes amongst patients on glucose-lowering medications.

Despite the breadth of work that has established metformin as an effective hyperglycemia treatment in diabetes during pregnancy, some studies have demonstrated that it has little benefit or even a detrimental effect on maternal and neonatal outcomes. In addition, there is evidence that metformin has a minimal effect on neonatal morbidity and mortality [84]. Additionally, although there is an overwhelming consensus that metformin is safe for use in the first trimester, biological changes in the placenta and fetus in the second and third trimesters may alter this. Barbour et al. suggested that during these trimesters, “metformin has the potential to inhibit mitochondrial activity and may adversely affect function, growth, or differentiation of fetal or placental tissues into which metformin is transported” [87]. Furthermore, it is possible that one of the most established effects of metformin—reducing maternal weight—is misleading in that it prevents weight gain rather than induces weight loss [86]. For these reasons, research continues in the field of antihyperglycemic agents, and novel approaches are being taken to manage pregnancies complicated by diabetes. Pettus et al., documented the differences in an array of glucose lowering agents currently in use or in development [88].

In reviewing the existing literature on diabetes management during pregnancy, certain key elements are identifiable when considering an ideal treatment. These include the early detection of GDM, commencing lifestyle intervention before pregnancy or in the early stages of pregnancy, using rapid-acting agents to achieve glycemic control, and engaging patients more effectively to increase compliance with intervention programs.

The first step in treating diabetes during pregnancy is early identification and intervention. Ideally, lifestyle intervention should begin before pregnancy, focusing on helping women achieve glycemic control and optimal pre-gravid weight. Achieving these targets early can prevent the development of GDM [35,37,42,51], reduce the likelihood of fetal hyperinsulinemia [89], decrease incidences of early induced labor and preterm births [38,45,90], normalize birth weights [38,91], prevent pre-eclampsia [90,91], and improve other maternal and neonatal outcomes. Despite this, a significant percentage of women with existing pregestational diabetes continue to not achieve good glycemic control in early pregnancy; a 2016 UK-based study found that 75% of women with T1DM and 64% of women with T2DM did not achieve target glucose levels in early pregnancy [92]. Another UK-based study found that only 17% of maternity clinics throughout the country offered pre-pregnancy care [93]. By expanding pre-pregnancy care programs and increasing outreach to women considering pregnancy, adverse outcomes of pregnancies affected by diabetes can be significantly reduced and care facilities may even cut costs due to the reduced need to manage such adverse outcomes [93]. The intervention strategies employed at this stage should focus on physical exercise, dietary planning, and the self-monitoring of blood glucose levels.

Physical exercise routines should aim for a minimum of 600 MET-min of moderate-intensity exercise per week, which reduces the odds of developing GDM and pre-eclampsia by 25% [94]. Surpassing this target can lead to more significant risk reductions [40,94], but proper compliance and verification of exercise goals will determine the effectiveness of this intervention. Studies examining the effects of multiple types of physical activity interventions on gestational diabetes and pregnancy outcomes have noted that although participants’ poor compliance may have skewed their studies’ results, they still experienced positive outcomes from an increase in baseline activity [42,94]. Given this, it has been suggested that the increased supervision and convenience afforded by telehealth services can enhance compliance and improve the results of physical exercise intervention [60].

Dietary intervention is another component of diabetes management that may benefit more from compliance than from an extremely precise plan. The DASH diet [94,95], ADA diet [49], and diets based on general DRI guidelines [50] have all been shown to improve glucose tolerance and reduce rates of cesarean section, as well as positively impact some neonatal outcomes. In a large-scale review of the effects of dietary intervention, defined as improved nutritional quality without a specific approach, on maternal and offspring outcomes in pregnancies complicated by GDM, Sweeting et al. found evidence of “improved maternal fasting and postprandial glycemia, and reduced pharmacotherapy requirements, birth weight, and macrosomia” that was “irrespective of the specific dietary approach” [48].

Where patients are unable to achieve glycemic control early in pregnancy and develop GDM or have existing diabetes before pregnancy, a targeted combination of lifestyle intervention and insulin treatment should be implemented to improve maternal and neonatal outcomes. Lifestyle intervention does not need to differ greatly from how it is approached pre-pregnancy, with obvious consideration given to the strenuousness of physical activity based on the stage of pregnancy. However, once GDM has been identified or in cases where pregnant mothers have pregestational T1/2DM, the use of continuous glucose monitoring systems should be incorporated. It is thought that increased self-scrutiny of glucose levels enhances compliance in other areas of intervention [62,63], and limited data have shown that the offspring of mothers using CGM systems have lower birth weight and decreased instances of macrosomia and hypoglycemia [63,96,97]. It appears that CGM systems have a more pronounced positive impact on glycemic control in pregnant women with GDM rather than in those with T1/2DM, though conflicting reports of this effect merit more robust investigation [63,96,97].

The major change in diabetes management that should be implemented once GDM has been identified or in the early stages of pregnancy complicated by T1/2DM is insulin therapy. As demonstrated in this work, metformin-based therapy is associated with improved maternal and neonatal outcomes in cases of pregnancy complicated by diabetes [48,72,77,78,79,80,82,84,85,86]. Although some studies have not found metformin outcomes to be significantly different from regular insulin treatment and other antihyperglycemic agents, there generally appear to be fewer adverse outcomes associated with metformin than with other commonly used therapies. In addition, metformin therapy is less likely to require supplementation with insulin therapy. From an outcomes perspective, supplementing metformin therapy with regular insulin has also been shown to significantly reduce the likelihood of cesarean delivery compared to either approach alone [84,85].

## 4. Section III: Areas for Future Research

Future work in this field should primarily focus on developing more effective glucose lowering agents, improving the quality and accessibility of telehealth options, and increasing pre-pregnancy identification and treatment options for women at risk of developing GDM.

The ideal therapy would be one that is completely unable to cross the placenta, unlike metformin and other therapies [98]. Overall, on review of the current literature at the time of publication, there is no such drug therapy that currently meets the parameters of the ideal therapeutic design described in this review. As Lefever and Mathieu concluded, current antihyperglycemic therapies still do not enable many patients to achieve glycemic targets and lead to hypoglycemia more frequently than desired [99]. Long-acting therapies, including insulin analogs, may be beneficial in establishing an optimal insulin control regimen for pregnant women with diabetes. However, there is currently insufficient evidence of their effectiveness in this context, with some studies actually indicating an increased incidence of neonatal hypoglycemia when being utilized [100]. One such analog, insulin glargine, has been shown to help pregnant patients improve their glycemic control and has little evidence of negatively impacting neonatal outcomes [88]. However, relatively few trials have investigated the impact of insulin glargine on pregnancy, and further research is necessary to confirm the safety and efficacy of this treatment [88]. Research on molecular and mechanical glucose-responsive insulin could revolutionize diabetes care, but these concepts remain largely theoretical at the moment [101]. Additionally, future investigation into automatic insulin delivery aided by continuous glucose monitoring would be beneficial for optimizing this therapy. Alternatively, artificial pancreas systems that address insulin needs have been designed and implemented, but they are still relatively novel and require further testing and development [101].

Innovation in telehealth is also poised to enhance outcomes for pregnancies complicated by diabetes. Effective telehealth care has consistently improved glycemic control in patients [102,103]. Through the utilization of technology, patients are now able to access care virtually, removing barriers to care that would exist if they otherwise were required to be in person. A telephone-delivered lifestyle intervention regimen developed by the Diabetes Prevention Program helped pregnant and postpartum women achieve or approach their pre-gravid weight and reduce dietary fat intake [104]. Telehealth-based care has also been shown to decrease the incidence of cesarean births among women with GDM [61,103]. Numerous studies have demonstrated that in addition to improving maternal outcomes, the use of telehealth methods in diabetes treatment increases overall patient satisfaction [105].

Furthermore, telehealth reduces access barriers that are common in rural areas [61,102,105]. McLendon emphasized that errors and long response times can reduce the effectiveness of this technology, making it essential to invest in training and supervising telehealth staff to maintain quality of care [105]. Additionally, improving patient retention strategies beyond reminder calls and mailings will enhance outcomes [102]. Xie et al. outline the effects of telemedicine interventions on maternal and fetal outcomes compared to those of standard care for patients with GDM [61].

When treating GDM and T1/T2DM during pregnancy, early intervention is crucial. Enhancing methods for screening GDM would also be beneficial. As Immanuel and Simmons discussed, accurate screening for GDM early is particularly challenging, as it encompasses four different phenotypes, and the natural variation in glycemic profiles throughout pregnancy complicates accurate detection [106,107]. There are many advocates for the International Association of the Diabetes and Pregnancy Study Groups screening method, with one study finding that 42% of women diagnosed using this criterion would have gone undetected if the Carpenter–Coustan method had been used [108]. Some have raised concerns about the risk of over-diagnosing GDM, but it has been difficult to link this to any adverse health outcomes beyond theoretical implications [91,109]. Multiple studies have shown that women screened and diagnosed with GDM are less likely to experience a range of adverse maternal outcomes, including hypertension and cesarean delivery [91,108]. Duran et al. found that improvements extended to offspring outcomes, including enhancing their one-minute APGAR scores [108]. However, it is noteworthy that other studies have found little difference in outcomes between patients who received early GDM screening and those whose GDM was identified later in pregnancy [110].

Moreover, some experts have cautioned that early screening may lead to an increase in erroneous GDM diagnoses, resulting in unnecessary procedures, higher costs, and more small-for-gestational-age infants due to overtreatment [106,110]. Ultimately, the best results are achieved when women with T1/T2DM or those predisposed to GDM begin treatment and lifestyle interventions before pregnancy. As screening methods improve over time, efforts to encourage the implementation of interventions prior to conception should be expanded, despite the inherent logistical challenges. Future work should leverage the globalization of the internet and smart technology to address these challenges with the development of web resources or an application that patients can utilize to assess their risk or need for health evaluation on their own time.

## 5. Summary

The global prevalence and subsequent disease burden of GDM continue to rise despite efforts to mitigate them through lifestyle changes and medical management. As abnormal gestational insulin resistance becomes more common, a greater proportion of mothers and their offspring face increased morbidity and mortality risks, not only during the gestational period but also throughout their lifespans. In this article, we analyzed the pathophysiological basis of insulin resistance in pregnancy and the downstream health effects of metabolic dysfunction, aiming to characterize the efficacy of current practice guidelines. Our review supports a combined therapeutic approach that includes the adoption of early initiation of lifestyle counseling on diet and exercise through optimizing telehealth interventions and the concurrent initiation of medical management with insulin therapy (first line) to improve treatment outcomes.

Further investigation into optimizing the timing for initiating screening for the disease and possibly beginning interventions in the preconception period is warranted. Additionally, further research into the development of safer and more effective antihyperglycemic agents, specifically therapies that do not cross the placenta, for use during pregnancy is indicated, as this would allow for earlier initiation of medical intervention.

## Abbreviation

ACC: Acetyl-CoA Carboxylase; ADA: American Diabetes Association; AMPK: 5′ Adenosine Monophosphate-Activated Protein Kinase; BMI: Body Mass Index; CGM: Continuous Glucose Monitor; DASH: Dietary Approaches to Stop Hypertension; DRI: Dietary Reference Intake; EGF: Epidermal Growth Factor; FAS: Fatty Acid Synthase; FFAs: Free Fatty Acids; GDM: Gestational Diabetes Mellitus; GSIS: Glucose-Stimulated Insulin Secretion; GWG: Gestational Weight Gain; hPGH: Human Placental Growth Hormone; hPL: Human Placental Lactogen; IADPCSG: International Association of the Diabetes and Pregnancy Study Groups; IGF: Insulin-Like Growth Factor; INS-R: Insulin Receptor; IRS-1: Insulin Receptor Substrate-I; LGA: Large for Gestational Age; LGI: Low Glycemic Index; LPL: Lipoprotein Lipase; MET: Metabolic Equivalent of Task; mTOR: Mechanistic Target of Rapamycin; PAI-1: Plasminogen-Activator Inhibitor-1; PI3K: Phosphatidylinositol-4,5-bisphosphate 3-kinase; PPARy: Peroxisome Proliferator-Activated Receptor Y; RDS: Respiratory Distress Syndrome; T1DM: Type 1 Diabetes; T2DM: Type 2 Diabetes; TAG: Triacylglycerides; TNF-α: Tumor Necrosis Factor; TSC2: Tuberous Sclerosis 2; UK: United Kingdom; VEGF: Vascular Endothelial Growth Factor.

## Figures and Tables

**Table 1:** Major factors contributing to a hyperglycemic state. The table categorizes important determinants and components of the hyperglycemic state.

Factors Contributing to Hyperglycemic State	Specific Component
Genetics	Insulin Receptor Transduction Efficacy
	Pancreatic Beta-Cell Function
Metabolic Conditions	Obesity
Inflammatory Factors	Autoimmune Conditions

## Data Availability

No new data were created or analyzed in this study.
